# The gut microbiome and type 2 diabetes status in the Multiethnic Cohort

**DOI:** 10.1371/journal.pone.0250855

**Published:** 2021-06-23

**Authors:** Gertraud Maskarinec, Phyllis Raquinio, Bruce S. Kristal, Veronica W. Setiawan, Lynne R. Wilkens, Adrian A. Franke, Unhee Lim, Loïc Le Marchand, Timothy W. Randolph, Johanna W. Lampe, Meredith A. J. Hullar

**Affiliations:** 1 Population Sciences in the Pacific, University of Hawai’i Cancer Center, Honolulu, Hawaii, United States of America; 2 Brigham and Women’s Hospital and Harvard Medical School, Boston, Massachusetts, United States of America; 3 Department of Preventive Medicine, University of Southern California, Los Angeles, California, United States of America; 4 Public Health Sciences Division, Fred Hutchinson Cancer Research Center, Seattle, Washington, United States of America; University of California Los Angeles, UNITED STATES

## Abstract

**Background:**

The gut microbiome may play a role in inflammation associated with type 2 diabetes (T2D) development. This cross-sectional study examined its relation with glycemic status within a subset of the Multiethnic Cohort (MEC) and estimated the association of circulating bacterial endotoxin (measured as plasma lipopolysaccharide-binding protein (LBP)) with T2D, which may be mediated by C-reactive protein (CRP).

**Methods:**

In 2013–16, cohort members from five ethnic groups completed clinic visits, questionnaires, and stool and blood collections. Participants with self-reported T2D and/or taking medication were considered T2D cases. Those with fasting glucose >125 and 100–125 mg/dL were classified as undiagnosed (UT2D) and pre-diabetes (PT2D) cases, respectively. We characterized the gut microbiome through 16S rRNA gene sequencing and measured plasma LBP and CRP by standard assays. Linear regression was applied to estimate associations of the gut microbiome community structure and LBP with T2D status adjusting for relevant confounders.

**Results:**

Among 1,702 participants (59.9–77.4 years), 735 (43%) were normoglycemic (NG), 506 (30%) PT2D, 154 (9%) UT2D, and 307 (18%) T2D. The Shannon diversity index decreased (p_trend_ = 0.05), while endotoxin, measured as LBP, increased (p_trend_ = 0.0003) from NG to T2D. Of 10 phyla, Actinobacteria (p_trend_ = 0.007), Firmicutes (p_trend_ = 0.003), and Synergistetes (p_trend_ = 0.02) were inversely associated and Lentisphaerae (p_trend_ = 0.01) was positively associated with T2D status. *Clostridium* sensu stricto 1, *Lachnospira*, and *Peptostreptococcaceae* were less, while *Escherichia-Shigella* and *Lachnospiraceae* were more abundant among T2D patients, but the associations with Actinobacteria, *Clostridium* sensu stricto 1, and *Escherichia-Shigella* may be due metformin use. PT2D/UT2D values were closer to NG than T2D. No indication was detected that CRP mediated the association of LBP with T2D.

**Conclusions:**

T2D but not PT2D/UT2D status was associated with lower abundance of SCFA-producing genera and a higher abundance of gram-negative endotoxin-producing bacteria suggesting that the gut microbiome may contribute to chronic systemic inflammation and T2D through bacterial translocation.

## Introduction

A role of the gut microbiome in glycemic control and type 2 diabetes (T2D) has emerged in recent years through several plausible biological mechanisms [1]. These potential mechanisms include adverse effects in the gut, transfer of lipopolysaccharide (LPS) into systemic circulation, either through “leaky” tight junctions or via chylomicron uptake, with subsequent systemic inflammation that can be assessed by circulating C-reactive protein (CRP) levels [2]. In contrast, gut bacterial production of short chain fatty acids (SCFA), including acetate and butyrate, in response to dietary fiber intake may prevent T2D through appetite control and energy homeostasis [3]. Additionally, butyrate has been shown to impact gut hypoxia by mitochondrial beta-oxidation and by interacting with HIF-1 alpha, a transcription factor, to maintain tight junctions [4, 5]. Substantial epidemiologic evidence also supports a relation between the gut microbiome and T2D. In several studies, individuals with T2D showed a lower abundance of butyrate-producing bacteria from the phylum Firmicutes and were more likely to host opportunistic pathogens [6–8]. For example, among 145 European women with T2D [9], *Roseburia* and *Faecalibacterium prausnitzii*, both known to produce butyrate and linked to improved insulin sensitivity, were identified as highly discriminant for T2D status. In addition, alpha diversity was lower among T2D patients than in individuals with normal glucose status, both in a Chinese study [6] and in a report from Mexico [10]. Interestingly, changes in the abundance and the function of gut microbiota after prescribing metformin for T2D treatment have been reported [3, 10, 11].

Taking advantage of a diverse population with a wide range of body weights and variation in glucose metabolism within the Adiposity Phenotype Study (APS), a sub-study of the Multiethnic Cohort (MEC), the current analysis addresses the hypothesis that individuals with abnormal glycemic status show different patterns of gut microbiome composition than normoglycemic persons. To evaluate a possible biologic mechanism [12], the possible role of CRP as a mediator of the association between lipopolysaccharide-binding protein (LBP) and T2D status was examined.

## Materials and methods

### Study population

This cross-sectional analysis was conducted within a subset of the MEC, which recruited more than 215,000 men and women aged 45–75 years at the time of cohort entry in 1993–1996. Five ethnic groups (white, African American, Native Hawaiian, Japanese American, and Latino) living in Hawai’i and Los Angeles were targeted to examine diet, lifestyle factors, genetics, and cancer risk and completed a 26-page questionnaire by mail [13]. During 2013–2016, 1,861 MEC participants aged 58–74 years of the same five ethnic groups and living in the catchment area of the study clinics were recruited into the APS, a cross-sectional study of the determinants of body fat amount and distribution [14]. The following exclusion criteria were applied: reported body mass index (BMI) outside the target range (18.5–40 kg/m^2^), current or recent (<2 years) smoking, soft or metal implants (other than knee or hip replacement) or amputations, claustrophobia, insulin treatment, thyroid medication, or other serious health conditions. Individuals with weight change of >9 kg or undergoing treatments or procedures that were likely to affect adiposity or biomarkers of interest, e.g., antibiotics, colonoscopy, chemotherapy, radiation of abdomen/pelvis, corticosteroids, weight loss drugs, estrogen/androgen receptor blockers, were deferred for 6 months, at which time their eligibility was reconsidered. Individuals with a T2D diagnosis requiring insulin were not eligible due to its possible effect on body fat distribution [15]. Through mailed invitations and screening telephone calls, eligible participants were enrolled within 60 sex/ethnicity/BMI strata (Table 1). The participation rate was 15.6% out of the 13,884 contacted, excluding the 4,455 persons who were willing but ineligible. Although the sample cannot be considered a fully representative sample of the cohort, the goal was to look at biological relations expected to be unrelated to reasons for participation. The University of Hawaii Biomedical Institutional Review Board (CHS# 17200) and the University of Southern California Biomedical Institutional Review Board (#HS-12-00623) approved the study protocols and all participants provided signed informed.

**Table 1:** Characteristics of the study population at clinic visit, APS, 2013–2016^a^.

Characteristic		All	NG^b^	PT2D	UT2D	T2D	p^c^
N (%)		1702 (100)	735 (43)	506 (30)	154 (9)	307 (18)	
Area, %	Hawai‘i	1085	38.8	35.7	11.1	14.4	
	Los Angeles	617	50.9	19.3	5.3	24.5	<0.0001
Sex, %	Men	844	37.2	33.2	9.8	19.8	
	Women	858	49.1	26.3	8.3	16.3	<0.0001
Ethnicity, %	White	383	47.5	37.1	9.4	6.0	
	African American	272	53.7	20.2	5.9	20.2	
	Native Hawaiian	277	38.3	32.5	10.1	19.1	
	Japanese American	423	31.4	36.2	13.5	18.9	
	Latino	347	48.4	19.0	4.9	27.7	<0.0001
Age at clinic visit, yrs		69.2±2.7	69.2±2.7	68.9±2.7	68.9±2.7	69.7±2.8	0.08
Fasting hours, hrs		13.9±2.1	14.1±2.2	13.6±2.1	13.3±2.1	14.1±2.1	<0.0001
Healthy Eating Index-2010		72.4±10.6	73.8±10.4	71.4±11.0	71.6±10.8	71.3±9.9	0.0001
BMI, kg/m^2^		27.9±4.8	27.2±4.7	27.9±4.8	28.0±4.8	29.6±4.7	<0.0001
DXA total body fat, kg		25.4±8.7	25.2±8.7	24.5±8.6	24.6±9.2	27.8±8.4	0.0002
Alcohol intake, %	<1 drink/mo	880	42.2	28.5	8.9	20.4	
	<1 drink/d	539	46.5	28.6	9.1	15.8	
	≥1 drink/d	283	39.9	35.7	9.6	14.8	0.06
Physical activity, %	<0.5 h/d	340	37.7	31.5	7.6	23.2	
	0.5 to <1 h/d	377	42.2	26.0	10.6	21.2	
	1 to <1.5 h/d	360	48.3	26.7	9.7	15.3	
	≥1.5 h/d	625	43.8	32.8	8.5	14.9	0.004
Biomarkers	CRP, mg/L	1.85±2.61	1.79±2.60	1.88±2.52	1.76±2.52	2.00±2.80	0.31
	LBP, μg/mL	22.7±8.2	21.9±8.4	22.7±7.8	22.9±8.5	24.6±7.9	<0.0001
	Glucose, mg/dL	108±29	88±9	110±7	141±22	137±41	<0.0001
	Insulin, μU/mL	7.12±4.79	5.95±4.04	7.27±4.59	8.78±5.85	8.86±5.36	<0.0001
	HOMA-IR	1.98±1.69	1.30±0.90	1.98±1.28	3.12±2.31	3.06±2.39	<0.0001

^a^Means ± standard deviations are shown unless otherwise indicated.

^b^NG = Normoglycemic; PT2D = Prediabetes (fasting glucose FG 100–125 mg/dL); UT2D = Undiagnosed T2D (FG >125 mg/dL); T2D = Self-reported T2D and/or taking diabetes medication.

^c^p-value of difference calculated from general linear models for continuous and chi-square tests for categorical variables.

### Data collection

In addition to questions about demographics, medical conditions, physical activity, and other lifestyle factors, all participants completed a quantitative food frequency questionnaire (QFFQ) containing over 180 food items [13]. As an overall assessment of diet quality, scores (0–100) were computed from the QFFQ data according to the Healthy Eating Index-2010 (HEI-2010), which reflects the 2010 Dietary Guidelines for Americans. Higher scores indicate better adherence to federal dietary guidelines [16]. As described in detail previously, APS participants visited study clinics to take part in anthropometric measurements, dual energy X-ray absorptiometry (DXA) and abdominal MRI imaging, fasting blood sample collection, and questionnaires [15].

### Microbiome analysis

Stool samples were collected in RNAlater^®^ and kept in the participants’ home freezer until the clinic visit when they were stored at -80°C. In a short questionnaire, participants reported antibiotic use during the past year. The samples were shipped on dry ice to the Fred Hutchinson Cancer Research Center for analysis [17]. DNA was extracted and amplified for the V1-V3 region of the 16S rRNA genes, and amplicons were sequenced on the MiSeq platform (Illumina, San Diego, CA). To classify bacterial taxonomy, sequences were processed using QIIME v.1.8 [18] and SILVA 1.32, as previously described [17]. The filtering strategy for operational taxonomic units (OTUs) included parameters in QIIME to exclude low abundant sequences, singletons, and chimeras and final filtering at the genera level, in which we removed genera which appeared in <10% of the samples [19]. More specifically, sequences were joined with the fastq-join method, using min_overlap = 15 and perc_max_diff = 12, then filtered with split_libraries_fastq.py with q parameter set to 25, and defaults otherwise. The Nelson two step method was used for OTU generation at 97% similarity using the SILVA database (release 132, clustered at the 97% similarity level) for the closed reference OTU picking step [20]. The OTU table was filtered using the QIIME script filter_otus_from_otu_table.py with—min_count_fraction set to 0.00005 [21]. An additional filtering step set entries in the OTU table to zero if the number of observations was less than 10 per-sample, per-OTU. Additional OTU entries were filtered out if they were detected as chimeras using QIIME’s identify_chimeric_seqs.py script with method blast_fragments [22–24]. Sequences were aligned to the Silva 16S rRNA gene reference using the NAST algorithm [23] and classified using MOTHUR’s naive Bayes classifier trained against the SILVA database [25]. The mean number of reads per sample was 34,148 (range: 9,152–169,013). Beta diversity was calculated using the Bray-Curtis distance metric on relative percent OTUs to adjust for differences in sequencing depth. ComBat-adjustment [26] to correct values across laboratory batches and centered log-ratio transformation (CLR) to account for their compositional nature [27] was applied to all phylum and genus variables.

### Lab assays

Venous blood (40 mL) was collected after an overnight fast (>8 hours), processed in the MEC laboratories in Hawaii and Los Angeles, and stored at -80°C until shipment for assays at the UH Cancer Center Analytical Biochemistry Shared Resource [28]. Samples were arranged into batches so that each batch included approximately equal numbers of men and women of each ethnic group and ~10% blind quality control duplicates. Insulin was assessed in serum using ELISA (EMD Millipore) and glucose by Cobas Mira Plus Chemistry autoanalyzer (Randox Laboratories). The Homeostatic Model Assessment of Insulin Resistance (HOMA-IR) was calculated as (fasting insulin (mU/L) × fasting glucose (mg/dL))/405. Plasma LBP was analyzed using ELISA (Cell Sciences; within and between batch variation coefficients of variation (CV): 4.3% and 11%, respectively; ICC 0.61–0.68). Serum high-sensitivity CRP was analyzed on a Cobas MiraPlus clinical chemistry analyzer (Roche Diagnostics, Indianapolis, IN) (Core Lab Supplies).

### Classification of glycemic status

Following the American Diabetes Association [29], participants with fasting glucose <100 mg/dL were classified as normoglycemic (NG), those with 100–125 mg/dL as prediabetes (PT2D), those with FG >125 mg/dL as undiagnosed diabetes (UT2D), and those with self-reported T2D or diabetes medication as T2D cases. As only 283 participants had Hb1Ac values, they were not used for classification.

### Statistical analysis

The SAS 9.4 (Cary, NC) package was used to perform all data analyses. Of the 1,861 APS participants, 78 were missing microbiome data, 34 did not have sufficient information to classify their T2D status, 34 had invalid dietary data, 21 did not have DXA information, and 6 were missing LBP values (some overlapping). After these exclusions, the final dataset for analysis had 1,702 observations.

Descriptive analyses assessed the differences across the four categories of NG, PT2D, UT2D, and T2D using chi-square tests for categorical and general linear models for continuous variables. To evaluate the association of microbiome characteristics with glycemic status as defined by the four categories, general multiple linear models were used to compute means for each glycemic category adjusted for age, sex, ethnicity, smoking status, alcohol use, physical activity, HEI-2010, DXA total body fat, antibiotic use, and 16S rRNA gene sequencing batch [30]. P-values of trend (p_trend_) across the four levels of glycemic status coded as discrete categories (0 = NG, 1 = PT2D, 2 = UT2D, and 3 = T2D) were computed by modeling glycemic status a continuous variable (0, 1, 2, and 3). Independent variables that were tested in separate models included three alpha diversity measures, as well as the relative abundance of 10 phyla and 152 genera. Beta diversity across categories of glycemic status was assessed using perMANOVA [31]. Bonferroni corrections were applied to the analysis of the 152 genera (0.05/152 = 0.00033) to decrease the likelihood of chance associations.

To assess differences in microbiome characteristics by metformin treatment, we divided the T2D group by medication use (no medication, other medication, and metformin) and modeled the five significant genera across all groups. Finally, we computed adjusted means and p-values (p_adj_) between T2D cases taking metformin (N = 202) vs. no diabetes medication (N = 67) with the same approach and covariates as above. The 38 participants who reported metformin in combination with another diabetes drug were excluded from this analysis.

Spearman correlation (r_s_) coefficients were computed for the correlation between LBP and CRP, the three diversity indices, all phyla, and the five significant genera considering a p-value of 0.05 as significant. LBP was assessed in relation to the four categories of T2D status using the same approach as described above. To examine if CRP, log-transformed to meet model assumptions, mediates the relation between LBP on T2D development [32], mediation modelling was performed through a comparison of beta coefficients for the LBP models without and with CRP.

## Results

Among the 1,702 participants (Table 1), the numbers of men (844) and women (858) were nearly equal; the mean age at clinic visit was 69.2±2.7 years. Overall, 307 participants (18%) were classified as T2D, 154 (9%) as UT2D, 506 (30%) as PT2D, and 735 (43%) were normoglycemic (NG). The proportion of NG was lowest among Japanese Americans (31%), followed by Native Hawaiians (38%), whites (47%), and Latinos (48%), and highest in African Americans (54%). Diet quality as assessed by HEI-2010 scores was 73.8 for NG participants and approximately 2 points lower for the other categories (p<0.0001). BMI and total DXA body fat were highest among T2D cases (29.6±4.7 kg/m^2^ and 27.8±8.4 kg).

As expected, fasting glucose and insulin levels increased across the continuum of glycemic status. Similarly, CRP and LBP values were significantly higher for T2D than NG participants.

All three alpha diversity indices (Table 2) were inversely related to glycemic status across categories (NG, PT2D, UT2D, T2D) and beta diversity was significantly different across glycemic status (p<0.001; S1 Table). For alpha diversity, the respective means were 6.28, 6.27, 6.28, and 6.17 (p_trend_ = 0.05) for the Shannon index and 18.2, 18.1, 18.4, and 17.6 (p_trend_ = 0.005) for PD whole tree, but the association was not significant for Chao 1 (p_trend_ = 0.10). Of 10 phyla, Actinobacteria (p_trend_ = 0.007), Firmicutes (p_trend_ = 0.003), and Synergistetes (p_trend_ = 0.02) were inversely related with T2D status while Lentisphaerae was positively associated (p_trend_ = 0.01). Of the 152 genera, only five (four Firmicutes and one Proteobacteria) were significantly associated with T2D status after Bonferroni adjustment (p_trend_≤0.00033). *Clostridium* sensu stricto 1 expressed as mean CLR values (-0.35, -0.43, -0.42, -0.89; p_trend_<0.0001), *Lachnospira* (0.22, 0.26, 0.25, -0.23; p_trend_ = 0.0003), and *Peptostreptococcaceae* uncultured (-0.47, -0.43, -0.26, -1.13; p_trend_<0.0001) were lower, while *Escherichia-Shigella* (0.24, 0.17, 0.30, 1.30; p_trend_<0.0001) and *Lachnospiraceae* uncultured (1.77, 1.72, 1.78, 2.00; p_trend_ = 0.0003) were more common among T2D than NG participants. In general, individuals with PT2D and UT2D status had values closer to those in the NG than in the T2D category. Splitting the T2D group by medication use (S1 Fig) indicated that metformin users had the most extreme values for the abundance of the five significant genera while those on no and other medications were closer to the UT2D group.

**Table 2:** Fecal microbial diversity and structure in relation to diabetes status, APS, 2013–2016^a^.

Characteristic	Variable	NG^b^	PT2D	UT2D	T2D	β	p_trend_^c^
N (%)	N = 1702	735 (43)	506 (30)	154 (9)	307 (18)		
Diversity	Shannon index	6.28	6.27	6.28	6.17	-0.031	0.05
	PD whole tree	18.2	18.1	18.4	17.6	-0.172	0.005
	Chao 1	661	660	669	647	-3.654	0.10
Phyla^d^	Actinobacteria	-0.27	-0.30	-0.27	-0.54	-0.078	0.007
	Bacteroidetes	4.20	4.24	4.10	4.12	-0.030	0.13
	Cyanobacteria	-0.65	-0.63	-0.63	-0.76	-0.029	0.44
	Firmicutes	4.49	4.54	4.40	4.30	-0.061	0.003
	Fusobacteria	-2.29	-2.37	-2.64	-2.03	0.050	0.29
	Lentisphaerae	-1.35	-1.29	-1.21	-1.05	0.096	0.01
	Proteobacteria	0.68	0.59	0.61	0.90	0.060	0.08
	Synergistetes	-1.17	-1.21	-1.36	-1.46	-0.098	0.02
	Tenericutes	-2.09	-2.20	-1.79	-2.02	0.044	0.36
	Verrucomicrobia	-1.55	-1.37	-1.21	-1.47	0.046	0.31
Genera^d^^,^ ^e^	*Clostridium sensu stricto 1*	-0.35	-0.43	-0.42	-0.89	-0.160	<0.0001
	*Escherichia-Shigella*	0.24	0.17	0.30	1.30	0.313	<0.0001
	*Lachnospira*	0.22	0.26	0.25	-0.23	-0.128	0.0003
	*Lachnospiraceae*; uncultured	1.77	1.72	1.78	2.00	0.069	0.0003
	*Peptostreptococcaceae*; uncultured	-0.47	-0.43	-0.26	-1.13	-0.176	<0.0001

^a^Obtained through general linear regression adjusted for age, sex, ethnicity, smoking status, physical activity, alcohol intake, DXA total body fat, antibiotic use, batch group, and HEI-2010.

^b^NG = Normoglycemic; PT2D = Prediabetes (fasting glucose FG 100–125 mg/dL); UT2D = Undiagnosed T2D (FG >125 mg/dL); T2D = Self-reported T2D and/or taking diabetes medication.

^c^P-value obtained from general linear model with diabetes status as continuous variable.

^d^ComBat-adjusted bacterial abundance variables after centered log-ratio transformation are shown.

^e^Five of 152 genera with significant trend after Bonferroni adjustment (0.05/152 = 0.00033).

Of the 307 participants with T2D (self-report or medication), 202 participants stated that metformin was their sole medication while 67 individuals did not report any diabetes medication (Table 3). Patients taking metformin had lower alpha diversity indices than those who did not: Shannon index (5.78 vs. 6.11; p_adj_ = 0.002), PD whole tree (16.7 vs. 18.0; p_adj_ = 0.001), and Chao 1 (612 vs. 648; p_adj_ = 0.02).

**Table 3:** Fecal microbial diversity and structure by metformin use, APS members with T2D, 2013–2016^a^.

Characteristic	Variable	Metformin		p_adj_^b^
		Yes	No	
Number^c^		202	67	
Diversity	Shannon index	5.78	6.11	0.002
	PD whole tree	16.7	18.0	0.001
	Chao 1	612	648	0.02
Phyla^d^	Actinobacteria	-0.99	-0.26	0.0005
	Bacteroidetes	4.24	4.01	0.10
	Cyanobacteria	-0.93	-0.78	0.57
	Firmicutes	4.19	4.22	0.25
	Fusobacteria	-1.67	-1.66	0.96
	Lentisphaerae	-0.55	-0.70	0.56
	Proteobacteria	0.81	0.40	0.05
	Synergistetes	-1.34	-1.46	0.68
	Tenericutes	-2.44	-2.00	0.18
	Verrucomicrobia	-1.34	-1.76	0.20
Genera in Actinobacteria	*Adlercreutzia*	-1.00	-1.18	0.29
	*Bifidobacterium*	-1.44	-0.93	0.03
	*Collinsella*	0.62	1.38	0.004
	*Coriobacteriales Incertae Sedis*; uncultured	-0.81	-0.95	0.45
	*Eggerthella*	-0.69	-0.72	0.87
	*Enterorhabdus*	-0.76	-0.41	0.12
	*Senegalimassilia*	-0.78	-0.73	0.76
	*Slackia*	-0.64	-0.51	0.47
Genera^d^^,^ ^e^	*Clostridium sensu stricto 1*	-0.72	-0.23	0.04
	*Escherichia-Shigella*	1.08	-0.36	<0.0001
	*Lachnospira*	-0.39	0.08	0.03
	*Lachnospiraceae*; uncultured	2.46	2.16	0.02
	*Peptostreptococcaceae*; uncultured	-1.79	-1.15	0.002

^**a**^Adjusted means obtained through general linear regression adjusted for age, sex, ethnicity, smoking status, physical activity, alcohol intake, DXA total body fat, antibiotic use, batch group, and HEI-2010.

^**b**^P-value of difference by metformin use obtained from general linear models.

^**c**^N = 38 reporting other diabetes medication were excluded.

^**d**^ComBat-adjusted bacterial abundance variables were used after centered log-ratio transformation.

^**e**^Five of 152 genera with significant trend after Bonferroni adjustment (0.05/152 = 0.00033).

Among the phyla, only Actinobacteria (-0.99 vs. -0.26) showed a significant inverse association with metformin use (p_adj_ = 0.005). Given the lower abundance of Actinobacteria among metformin users, we examined the eight individual genera within this phylum. Of these, only *Bifidobacterium* (p_adj_ = 0.03) and *Collinsella* (p_adj_ = 0.004) were less abundant in participants reporting metformin use. Of the five genera associated with T2D status, only *Escherichia-Shigella* (1.08 among metformin users vs. -0.36; p_adj_<0.0001) met a Bonferroni-corrected significance level. Otherwise, *Lachnospiraceae* uncultured (2.46 vs. 2.16; p_adj_ = 0.002) were higher in individuals taking metformin, while *Clostridium sensu stricto 1* (-0.72 vs. -0.23; p_adj_ = 0.04), *Lachnospira* (-0.39 vs. 0.08; p_adj_ = 0.03), and *Peptostreptococcaceae* uncultured (-1.79 vs. -1.15; p_adj_ = 0.002) were lower. CRP levels between T2D patients taking and not taking metformin were not statistically significantly different (2.00 vs. 1.93; p_adj_ = 0.85).

LBP was significantly correlated to CRP (r_s_ = 0.37; p<0.0001), two phyla: Lentisphaerae (r_s_ = 0.05; p = 0.05) and Proteobacteria (r_s_ = 0.06; p = 0.02), two genera: *Escherichia-Shigella* (r_s_ = 0.06; p = 0.01) and *Peptostreptococcaceae*; uncultured (r_s_ = -0.05; p = 0.04), but not to any of the diversity indices. Mean LBP levels were associated with T2D status across glycemic status categories (23.2, 24.2, 24.3, 25.4.0 μg/mL for NG, PT2D, UT2D, T2D) with a significant trend across categories (p_trend_<0.0001). When CRP was included in the LBP model to test its role as a potential mediator between LBP and T2D status, the respective regression coefficients without and with CRP in the LBP model were 0.0133 and 0.0144, which provides no indication of CRP mediation on the relationship between LBP and T2D status.

## Discussion

This cross-sectional analysis detected a number of noteworthy associations between characteristics of the gut microbiome and T2D status. When we compared the alpha diversity indices along a "continuum" from NG to PT2D, UT2D and T2D, the observed decline was expected according to our hypothesis. Compared to NG participants, the abundance of Actinobacteria and Firmicutes, including three genera from this phylum, was lower in T2D cases. One genus each from the phyla Firmicutes and Proteobacteria were significantly more abundant in T2D cases than the other groups. The 10% higher LBP levels among T2D cases than NG participants might offer a biologic mechanism by which differences in the gut microbiome adversely affect metabolic status, although the minor difference may not be clinically relevant. This analysis did not find evidence for a mediation effect of CRP between LBP and glycemic status, but other pathways may explain how LBP may be linked to T2D etiology. Among T2D patients not taking metformin, alpha diversity was higher and the abundance of Actinobacteria including the genera *Bifidobacterium* and *Collinsella* was lower than in those taking metformin raising the question whether the associations of specific bacteria with T2D might be induced by medication.

The significant inverse association of alpha diversity with T2D and metformin use after adjusting for total adiposity are in agreement with previous reports of lower alpha diversity levels related to the development of glucose intolerance [6, 7, 33]. Lower alpha diversity has been described from studies in China [6], Germany [34], Pakistan [35], and Mexico [10] where values were only low for untreated T2D cases and those on metformin but not for those with polypharmacy therapy. However, null findings [7, 8, 36, 37] and positive relations with T2D [38, 39] were also reported for alpha diversity. In terms of diversity, metformin users in our study had a less favorable gut microbiome profile than participants reporting T2D and no medication. These findings do not agree with previous reports, possibly due to confounding by different medications taken and the fact that the UT2D groups was untreated. The higher alpha diversity in UT2D than T2D participants is probably a result of metformin treatment. Interestingly, a report from Mexico [10] indicated that the gut microbiome in T2D patients taking metformin was more similar to those taking no medication than patients taking many medications.

Given that Firmicutes is the phylum with the greatest number of genera in the gut and that it encompasses a broad range of metabolic diversity, the inverse association of four genera from this phylum with T2D status in our study is not a surprise. Three previous reports agree with lower abundance of Firmicutes by T2D status: a study from Denmark [8], one from Mexico [10], and one from Nigeria [39], while a higher abundance was reported from Pakistan [35] for obese individuals with T2D and from India [38] with elevated abundance primarily seen in undiagnosed T2D cases. Many of the other analyses reported on individual members within the class of anaerobic *Clostridiales* of the phylum Firmicutes and observed significantly higher [9, 35] or lower [7, 8, 39, 40] abundance among T2D cases. For example, among 145 European women with normal, impaired or diabetic glucose control [9], the metagenomic clusters (MGCs) most significantly enriched in T2D women were *Clostridiales* as well as *Lactobacillus gasseri* and *Streptococcus mutans* [41]. Twenty-one MGCs were significantly depleted in T2D, including *Roseburia*, *Clostridiales*, *Eubacterium eligens*, *Coriobacteriaceae*, and *Bacteroides intestinalis* [42]. Many of these organisms are associated with dietary fiber metabolism and butyrate production, such as *Roseburia* [43]. When obesity and treated T2D cases were investigated among a German population [34], obesity was associated with more alterations in microbiome composition, individual taxa, and functions than T2D status.

In our study, bacteria that ferment fiber to SCFA appear to be depleted in T2D but not in PT2D and UT2D. Bacteria-derived butyrate, a SCFA, helps to maintain physiologic hypoxia in the colon through several mechanisms including mitochondrial beta-oxidation in gut epithelium and interaction with hypoxia inducible factor 1 (HIF1A) which regulates oxygen potential to maintain tight junctions and gut epithelial integrity [4, 5]. Disruption of these pathways may affect host health. Possible explanations for the variable trend in the abundance of butyrate-producing bacteria in our study are the influence of metformin or other medications on the gut microbiome [44, 45] or confounding by obesity [7, 9, 45]. As shown recently, a health-supporting eating pattern was associated with the presence of *Lachnospira* in the Malmo offspring study [46] probably due to its ability to ferment diverse plant polysaccharides to SCFAs. The significant inverse associations of T2D status with three genera of the phylum Firmicutes are in agreement with this hypothesis. *Clostridium* sensu stricto 1 [47], one of the most important anaerobes in the gut, *Lachnospira*, and *Peptostreptococcaceae* all belong to the order *Clostridiales*, which metabolize carbohydrates and amino acids to produce butyrate through fermentation [47–50].

Metabolic endotoxemia is a persistent, low-grade, systemic inflammation associated with obesity and metabolic disease [51–53]. Greater abundance of gram-negative, endotoxin-producing bacteria in the *Escherichia-Shigella* group (which includes *E*. *coli*) [7, 34, 38] and other *Proteobacteria* [8, 39] among T2D patients have been reported previously. Here, we found a combination of a positive trend in LBP (a biomarker for endotoxin), endotoxin-producing bacteria from the *Escherichia/Shigella* group and a reduction in butyrate-producing genera. Metabolic endotoxemia may be enhanced when the endotoxin produced by the *Escherichia-Shigella* group is translocated across the weakened gut epithelium into circulation, forms complexes with LBP and TLR4, and triggers an inflammation cascade through the NFkB pathway [51, 53–55]. As has been shown previously [56], gram-negative bacteria may mediate systemic inflammation in obesity. Our data support the idea that this mechanism may operate across a gradient of metabolic sequelae and the T2D disease gradient [51–53, 57, 58].

We found that two Actinobacteria, *Collinsella*, and *Bifidobacteria*, were significantly reduced in metformin users. *Collinsella*, a member of the *Coriobacteriaceae* family has been positively associated with insulin, C-peptide and HOMA-IR [59]. Our data supports other studies that suggest members of the family *Coriobacteriaceae* may be markers of obesity associated with altered lipid and bile acid metabolism in metabolic syndrome [6, 33]. For example, strains of *Collinsella* are involved in bile acid transformations via hydroxysteroid dehydrogenase activity, which is altered in T2D [60]. The phylum Actinobacteria [38, 39] or specific genera, such as *Bifidobacterium* [36] or *Eggerthella*, a member of the *Coriobacteriaceae* family [7], were enriched among T2D cases in previous reports.

An emerging hypothesis suggests that part of the glucose-lowering effect of metformin is related to the gut microbiome and that metformin even decreases colorectal cancer risk among patients with T2D [61]. Early work showed that in contrast to oral dosing, the efficacy of metformin to control hyperglycemia was reduced when administered intravenously and suggested that the intestine was an important site of action for metformin [62]. Taking metformin was associated with a change in the composition of the fecal microbiome in subjects with T2D [45], including enrichment in *Escherichia* and reduction in *Intestinibacter* (Family *Peptostreptococcaceae*) [11, 45]. We also found that the microbiome of participants taking metformin was enriched in *Escherichia-Shigella* and depleted in an uncultured member of the *Peptostreptococcaceae*. In persons with T2D, enrichment of *Escherichia* is often associated with inflammation or metabolic endotoxemia, whereas metformin users show no increase in systemic inflammation [63], a finding that was corroborated in our study. Recent studies suggest that metformin reduces systemic inflammation by improving gut mucosal integrity via the promotion of the expression and assembly of tight junctions in an AMPK-dependent way [64, 65]. This mechanism may also impact Map3 kinase activation, a prognostic marker altered in CRC [66].

The current analysis has a number of strengths: foremost, the multiethnic population with a relatively high prevalence of T2D and a wide range of BMI; the established and routinely used microbiome analysis; and the detailed information collected about study participants including DXA measures and biomarkers. On the other hand, the one-time measurement of FG without HbA1c assessment limited our ability to classify participants with undiagnosed T2D. As detailed information on the duration and severity of T2D was not available, misclassification of the three categories may have occurred. The cross-sectional design does not allow for a causal interpretation, i.e., the differences in the gut microbiome characteristics may be the result of existing glucose abnormalities or T2D and not contribute to the development of T2D. Our study represents one of the largest studies of gut microbiome and diabetes although the number of T2D patients not taking metformin was quite small and limited our ability to draw conclusions from the comparison for patients not taking metformin. Indeed, as the majority of T2D patients were taking metformin and as indicated by the patterns after dividing the T2D group by medication use (S1 Fig), it appears possible that the differences in microbial composition by T2D status were driven by metformin. However, the number of T2D patients not taking metformin was very small. Differences to other studies may include geographic differences in the prevalence of bacteria structure [67]. However, the adjustment by ethnic group are expected to control for area, as African Americans and Latinos represent California and the other three groups Hawaii. Varying sequencing methods for 16S rRNA gene analysis and metagenomics may have also led to contradictory findings across reports [6–10, 34–36, 40, 68]. Our study would benefit from metagenomic functional gene sequencing and metabolic modeling analysis to understand the association between microbial and host metabolism as functionality may mirror genera identified using 16S rRNA gene analysis. Although ComBat-adjustment was applied to account for batch effects, some residual in prevalence and abundance of bacteria was probably not corrected. For example, for the 100% prevalent *Bacteroides*, this adjustment method is appropriate but, for less prevalent bacteria like *Faecalitalea*, it is less effective as ComBat does not have a zero inflated aspect to the model.

In this analysis from a multiethnic study, participants with T2D showed lower alpha diversity, a lower abundance of bacteria capable of fermenting plant polysaccharides, and higher levels of gram-negative endotoxin-producing bacteria. These findings support the hypothesis that a less favorable pattern of microbiome community structure in the gut due to dietary and other exposures may contribute to T2D through endotoxin binding to toll-like receptors via LBP and activation of the NFkB pathway associated with chronic systemic inflammation. Alternatively, individuals with abnormal glucose metabolism may experience disturbed microbiome patterns due to their condition. This question of causality can only be clarified using a longitudinal study design where the microbiome and metabolites are analyzed repeatedly before and after onset of T2D [69]. If the microbiome can be confirmed as a contributor to disease development, an integrated analysis approach of combining the microbiome with other risk factors would be indicated to develop future prevention strategies.

## Supporting information

S1 Table perMANOVA of beta diversity of the microbiome by diabetes status using Bray-Curtis distance metric. (DOCX)

S1 Fig Beta diversity of the microbiome color-coded by diabetes category: Bray Curtis distance metric was used to calculate the dissimilarity matrix followed by PCOA analysis, APS, 2013–2016. (TIF)

S2 Fig Genera in relation to diabetes status by medication use, APS, 2013-2016^a^. (TIF)
