# Supplementary material for: The gut microbiome and type 2 diabetes status in the Multiethnic Cohort
Source: PLoS One. 2021 Jun 23;16(6):e0250855. doi: 10.1371/journal.pone.0250855 (PMC8221508; doi:10.1371/journal.pone.0250855)
Supplement: S1 Table — (DOCX) [file pone.0250855.s001.docx]

|  |  |  |  |  |  |  |
| --- | --- | --- | --- | --- | --- | --- |
|  |  |  |  |  |  |  |
| S1 Table. perMANOVA of Beta Diversity of the Microbiome by Diabetes Status using Bray-Curtis Distance Metric, APS 2013-2016 | | | | | | |
|  |  |  |  |  |  |  |
|  | Df | SumsOfSqs | MeanSqs | F .Model | R^2^ | Pr (>F) |
| Diabetes Status | 3 | 1.708 | 0.569 | 2.050 | 0.004 | 0.001 |
| Residuals | 1698 | 471.588 | 0.278 |  | 0.996 |  |
| Total | 1701 | 473.296 |  |  | 1.000 |  |
|  |  |  |  |  |  |  |
