# Supplementary figures and images for: The gut microbiome and type 2 diabetes status in the Multiethnic Cohort
Source: PLoS One. 2021 Jun 23;16(6):e0250855. doi: 10.1371/journal.pone.0250855 (PMC8221508; doi:10.1371/journal.pone.0250855)

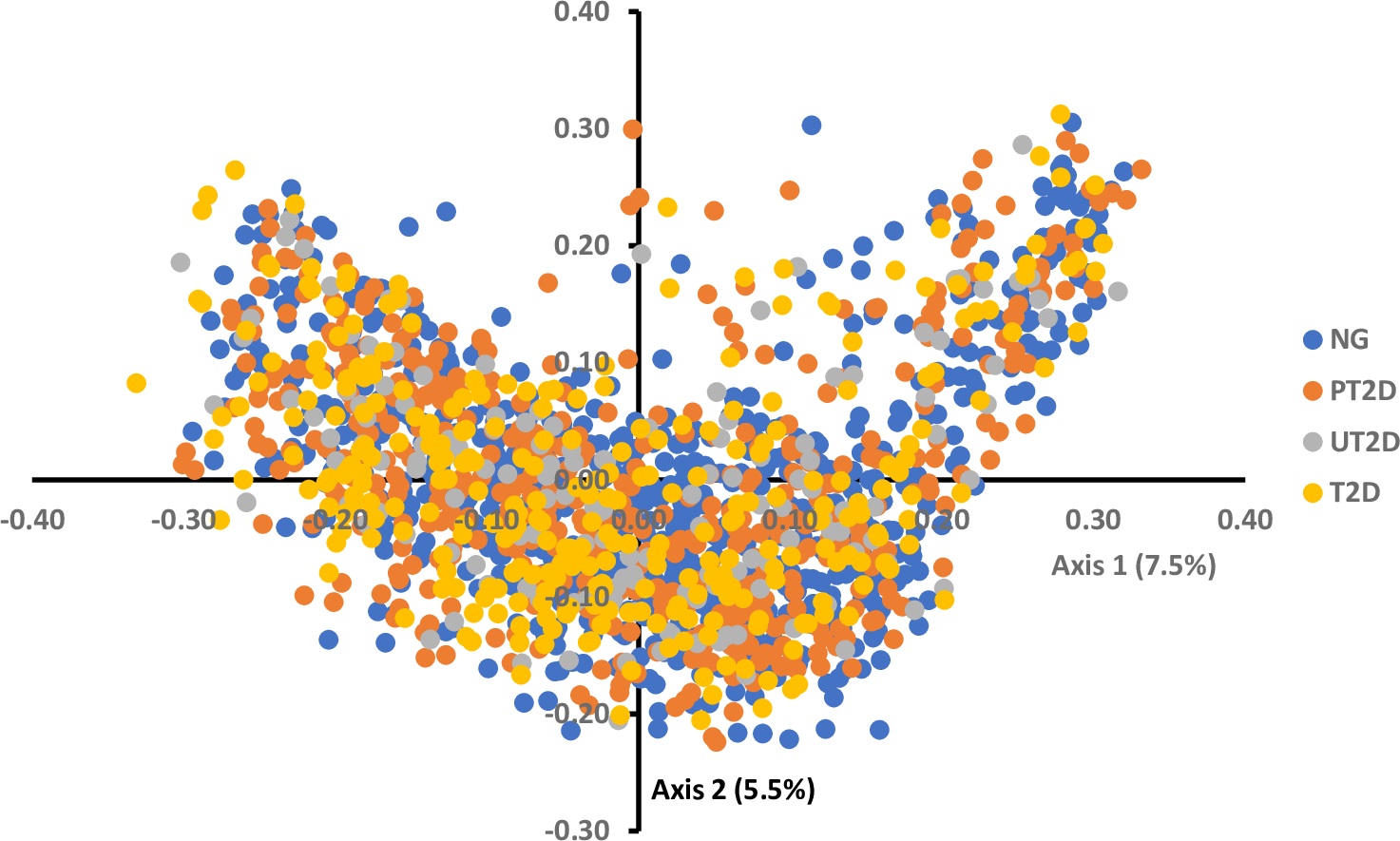

Supplement: S1 Fig — (TIF) [file pone.0250855.s002.tif]

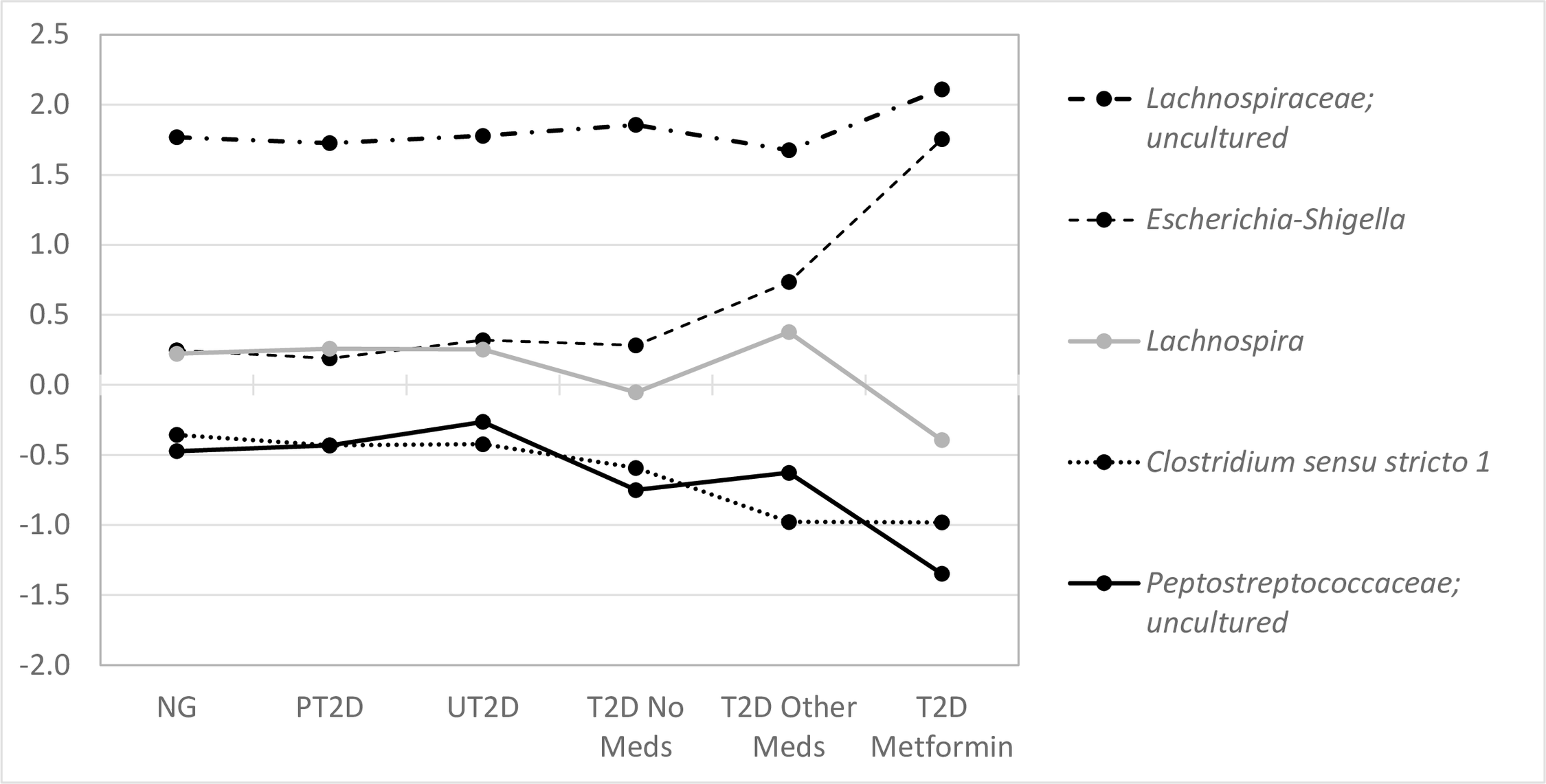

Supplement: S2 Fig — (TIF) [file pone.0250855.s003.tif]
